# Consumer Attitudes and Acceptability of Wheat Pancakes with the Addition of Edible Insects: Mealworm (*Tenebrio molitor*), Buffalo Worm (*Alphitobius diaperinus*), and Cricket (*Acheta domesticus*)

**DOI:** 10.3390/foods12010001

**Published:** 2022-12-20

**Authors:** Aleksandra Mazurek, Agnieszka Palka, Magdalena Skotnicka, Stanisław Kowalski

**Affiliations:** 1 Department of Commodity Science, Faculty of Health Sciences with the Institute of Maritime and Tropical Medicine, Medical University of Gdańsk, 80-210 Gdańsk, Poland; 2 Department of Quality Management, Faculty of Management and Quality Science, Gdynia Maritime University, 81-225 Gdynia, Poland; 3 Department of Carbohydrate Technology and Cereal Processing, Faculty of Food Technology, University of Agriculture in Krakow, Al. Mickiewicza 21, 31-120 Kraków, Poland

**Keywords:** food based on edible insect, insect meal, pancakes, acceptability

## Abstract

The aim of this study was to determine the degree of acceptability of wheat pancakes with the addition of 10%, 20%, and 30% meal from three edible insect species (*Alphitobius diaperinus*, *Tenebrio molitor, Acheta domesticus*, respectively). Both consumer attitudes and the acceptability of the test samples were analysed. The study results show that the amount of additive had a statistically significant effect on all of the organoleptic evaluation’s distinguishing features, while the type of additive did not have such a significant effect on the level of consumer acceptability. Both the type and amount of the additive only had a statistically significant effect on the structure of the pancakes. Of all the variants, the sample with the addition of 30% crickets (Pref-2.51) was given the lowest score. An increase in the insect meal content of the products resulted in decreased scores for all the parameters under assessment. The key element that influenced the overall preference was the flavour. Even though the respondents declared positive attitudes towards the idea of consuming pancakes with the addition of insects and entomophagy in general, they were still reluctant to include insects in their diets.

## 1. Introduction

The reluctance to consume insects (or to entomophagy) in Western cultures is a very common phenomenon [1,2]. This is most often related to concerns about the product flavour, aroma, and structure, as well as health safety [3]. As demonstrated in a recent study by Ardoin and Prinyawiwatkul, the willingness to eat insects was closely linked to the product and the form in which it was to be served [4]. Consumers declared their willingness to try to eat insects as an ingredient of commonly enjoyed foods, such as cereal products, sweets, protein cocktails, and meat analogues [5,6,7]. Products familiar to consumers that are widely accepted and associated with positive feelings are vehicles for enrichment with insects, which are achieving high acceptability. The use of insects offsets the adverse effects of food neophobia and a low level of knowledge about entomophagy on the general willingness to consume insects. Therefore, the elements of consumer readiness for insect consumption include their state of knowledge and previous experiences [8]. However, the key issues continue to be sensory qualities, including the form in which the insects are served. In a powdered form, they are gaining increasing acceptability [9]. In the USA, there was much greater support for foods with the addition of insect meal than for foods with whole insects added. Furthermore, the most common reason for rejecting the potential consumption of insects included disgust (Americans 57%, Indians 38%), followed by neophobia (19% and 17%, respectively), and sensory aversion (12% and 31%, respectively) [10]. Sensory qualities are a decisive determinant of the acceptability of insects, irrespective of country. On the other hand, the form of the insects and the way they are served were crucial for acceptability among the citizens of Western-culture countries [11]. Depending on the individual inclination towards seeking culinary sensations, the consumer is able to either accept or reject a new food product. Anxiety and concerns about new foods are referred to as food neophobia and are part of a tendency towards avoiding unfamiliar foods, influenced to a large extent by the native culture. Neophobic attitudes towards various products and dishes are determined inter alia by the diet considered traditional by the particular individual. Different products will cause food neophobia in different ethnic and social groups. For this reason, the level of neophobia towards insect consumption will be different in Western countries from that in the countries where entomophagy is known and widespread [12]. Negative attitudes towards unfamiliar foods result from a lack of knowledge, existing stereotypes, or influences from the community. They are, however, a very strong predictor of behaviours. Quite often, as a result of these factors, it is impossible to have a positive experience associated with foods containing edible insects.

Grain-based nutrition has long been a dietary staple for many cultures worldwide and is the most important source of nutrients for humans. Cereal products are an assortment group of products that are very commonly enriched with functional ingredients. One of the aims of food improvement is to enhance the nutritional value of a product. Regarding edible insects, the enrichment of cereal products is primarily aimed at increasing the amount of highly digestible proteins [13]. On the other hand, it may be of importance for carbohydrate-restricted diets or reduction diets. As for diets aimed at reducing body weight, protein is a desirable dietary component due to its very high satiating potential [14,15]. Insect meat contains all the essential amino acids, in particular lysine, tryptophan, and threonine. What is more, the digestibility of insect protein averages 76–98%, which is higher than that of peanuts or lentils and only slightly lower than that of beef or egg protein [15]. Furthermore, insects provide food with a high vitamin and mineral content [16]. An additional advantage of enriching products with insect meal is the high mineral and bioactive compound content [17,18]. These properties make edible insects a valuable food ingredient that can be used to design functional foods with a very wide range of applications. A review by Ros-Baró et al. showed that insect supplementation reduced abdominal and epididymal fat weight, blood glucose, total cholesterol, and triglycerides levels, and reduced body weight. Additionally, including insects in a diet could increase microbiota diversity [19]. Insect protein is characterised by a slower rate of digestion than soy and whey isolates. Unlike soy and whey isolates, insect protein isolate does not cause a large increase in insulin secretion [20]. This is beneficial considering the increasing prevalence of abnormal carbohydrate metabolism and metabolic syndromes [21]. The consumption of insects may contribute to the prevention of diseases in highly developed countries.

The dissemination of insects in nutrition also has widespread economic and environmental benefits. Large-scale insect farming generates less waste. Moreover, the biomass conversion rate is lower and the production time is much shorter than that for any other animal, and the water and land consumption is lower than that for conventional breeding [22]. The low environmental cost of insect protein is one of its main advantages compared with other protein sources [23]. Additionally, certain insects, e.g., *T. molitor*, are omnivorous and adapted to feeding on waste. The development of insect farming can also contribute to reducing the problem of the disposal of some waste [24,25].

With the above facts in mind, the aim of this study was to assess consumer attitudes towards insect-based foods, analyse the acceptability of pancakes prepared with the addition of three insect species, and try to determine the individual sensory distinguishing features affecting the testers’ overall acceptability. Currently, there are no published literature data on the enrichment of pancakes with edible insects. The results of the current study will enable the assessment of consumer attitudes and acceptance towards new products.

## 2. Materials and Methods

### 2.1. Pancake Preparation

This study used three insect species, i.e., mealworms *(Tenebrio molitor)*, buffalo worms *(Alphitobius diaperinus)*, and crickets *(Acheta domesticus).* The *A. diaperinus* and the *T. molitor* were in the larval form, while the *A. domesticus* were in the imago form. Lyophilised insects were sourced from the Netherlands from the farm Insecten kwekrij van de Vn Fortweg, Deurne. Whole insects in the lyophilised form were ground in a laboratory mill (IKA, A11 basic, Germany), and the pancakes were prepared in three variants. The introduced modifications involved the substitution of wheat meal with 10%, 20%, and 30% insect meal. The control pancake sample was prepared with no addition of insect meal. The exact composition of pancakes is provided in Table 1.

### 2.2. Nutrient Composition of Pancakes Enriched with Insect Meals

The pancakes with the addition of insect meals were analysed in relation to the control sample in terms of the ash, fat, protein, and water contents using standard analytical methods [26] and taking into account the conversion coefficient appropriate for insect protein (6.25). Carbohydrates per 100 g were determined based on the difference according to the following formula:

Carbohydrates = [100 − (weight in grams (protein + fat + ash + water contents, fibre)](1)

### 2.3. Colour Analysis

Colour measurements of pancakes were carried out using the CIE Lab system. The obtained results were expressed in terms of CIE L*, a*, and b* values. L* indicates brightness, a* represents red to green coordinates, and b* represents the blue to yellow coordinates of a product [CIE DS 014-4.3/E:2007]. The colour of the pancakes was determined using a Konica Minolta CM-5 spectrophotometer (Konica Minolta Sensing, Osaka, Japan). The measurement angle was 10°, and a D65 illuminator with a diaphragm of 8 mm was used. Measurements were made at 10 different locations on the surface of the pancakes. Measurements were made on the pancakes immediately after cooling them to room temperature. Average colour parameters were determined, and the total colour difference (in relation to pancakes prepared from wheat flour) was calculated from the formula [27]:

$$∆E=\sqrt{∆L^{2}+∆a^{2}∆b^{2}}$$

where

∆L=brightnes difference

∆a=reness difference

∆b=yellowness difference

In the analysis of the results, a criterion was used, according to which the absolute colour differences (ΔE*) between 0 and 1 are unrecognizable (invisible deviation); from 1 to 2 show a slight deviation, recognizable by a person experienced in distinguishing colour nuances; from 2 to 3.5 show a mean deviation recognised even by an outsider; from 3.5 to 5 show a clear deviation; and ΔE * above 5 means a large colour deviation. The above data are statistical, experimentally proven, and commonly used.

Based on L*a*b* parameters, the browning index (*BI*) was estimated [28,29,30,31,32] as follows:

*BI* = [100(*X* − 0.31)]/0.17194

where:

*X* = (*a*∗ + 1.75*L*∗)/(5.645*L*∗ + *a*∗ − 3.012*b*∗)

Measurements were performed in twelve repetitions.

### 2.4. Acceptability of Insect-Based Pancakes

The pancakes were served to testers immediately after cooling. The study involved 60 participants aged from 19 to 23 years old, who were selected from the volunteer database of the Medical University of Gdańsk. The acceptability test was conducted every other day for each pancake variant. Each participant tested all the samples by using 10 cm visual unstructured scales. The study was conducted as a triple-blind study. The respondents were healthy, took no medications or supplements, and had no special diets. All the respondents signed a voluntary consent form for participation in the study, which was approved by the Independent Institutional Ethics Committee for Scientific Research at the Medical University of Gdańsk (NKBBN/346/2021). Before the study, all participants were subjected to a food neophobia assessment according to the Food Neophobia Scale (FNS) [33]. Only individuals who exhibited no food neophobia participated in the study. The appearance, aroma, flavour, structure, and overall preference were rated using visual scales with the extreme indications of “Totally not to my liking” and “Totally to my liking” [34].

### 2.5. A survey on Attitudes towards the Consumption of Pancakes with Insect Meal Added

In addition to the organoleptic evaluation, a survey on consumer attitudes towards edible insects was conducted. The questionnaire contained a set of statements scored on a 5-point Likert scale, ranging from “strongly disagree” to “strongly agree”. The responses were assigned a number of points corresponding to the increasing intensity of a particular characteristic. For the statements containing negation, the scoring was reversed. A greater number of points indicated a more positive attitude towards the consumption of insects. The questionnaire contained seven questions, including five positive and two negative ones, in relation to the products with insects added being consumed. Responses 4, “I rather agree”, and 5, “I strongly agree”, were qualified as positive attitudes toward the consumption of insects. For questions 1 and 3, the reverse scoring was used, as they contained negative questions. Response 3, “It is difficult to say”, was qualified as an ambivalent attitude. Responses 1, “I strongly disagree”, and 2, “I rather disagree”, were qualified as negative attitudes [35]. The questionnaire was completed immediately after the consumption of pancakes and included the following statements (Table 2):

### 2.6. Statistical Analysis

Two-way analysis of variance (ANOVA) was used for statistical analysis. The type of meal, percentage of substitution, and their interactions were considered. Calculations were made using Microsoft Office Excel 2016 16.0.15831.20098 version 2211 and Statistica 13 1984-2017 TIBCO software “StatSoft, Poland”. The post hoc Tukey’s procedure was used to find patterns and relationships between subgroups. Differences among groups were determined as statistically significant at a level of *p* ≤ 0.05.

The results are presented as the mean value and standard deviation. At the experiment planning stage, the selection of the sample size at a level that provided statistical conclusions with adequate accuracy and confidence, and the probability of the detection of the effects of the given size by the test, were investigated based on test power analysis and interval estimation. The parameters of multiple regression, taking into account the concept of shared variability, were estimated using the REGLINP command in an Excel 2010 PL spreadsheet.

The results were analysed using a one-way analysis of variance (ANOVA). A *t*-test was applied to compare the mean values, and a *p*-value of 0.05 was used to determine significant differences. Pancake texture was tested on a Brookfield CT3 using texture profile analysis (TPA). The pancake texture was measured over a 25 min period immediately after cooking while the pancake was cooling. A flat (2.54 cm) acrylic cylinder was used in a double-bite compression test with a 5 g trigger point. The probe compressed the sample at 100 mm/min test speed until a 50% deformation target was reached. The following TPA parameters were measured: hardness and cohesiveness [31,32]. The measurements were performed in nine repetitions.

## 3. Results and Discussion

### 3.1. Nutritional Value of Lyophilised Edible Insects

For each insect species, the addition of insect meal increased the protein, fat, and fibre contents. The greatest increase in the protein and fibre contents in the composition of pancakes was noted for the variant with the addition of 30% *A. domesticus* meal. The highest energy value was exhibited by *T. molitor* meal, which is also rich in fat. Meals from *T. molitor* and *A. diaperinus* were characterised by a higher fat content than that in the meal from *A. domesticus*. This is due to the fact that, before grinding, *A. domesticus* were in the imago form, while *T. molitor* and *A. diparerinus* were in their larval form. The imago form is another factor that caused the protein and fibre (chitin) content to be higher than that for the larvae [15]. The nutritional values of the pancakes are provided in Table 3.

On the basis of the obtained results, it can be concluded that insect flour is a factor that statistically significantly increased the protein content in the pancakes. However, according to Janssen et al. (2017a), the conversion factor used to convert nitrogen to protein was too high. The authors indicated the necessity to individually select the conversion factor separately for each insect species and indicate the presence of different indigestible nitrogen contents at different development stages within one insect species [36]. For the larval forms of insects, Janssen et al. (2017b) suggest a conversion factor of 4.75 for *T. molitor* and 4.86 for *A. diaperinus* [37]. In the available literature data, there is currently no proposal of a conversion factor appropriate for the imago of *A. domesticus*. A separate issue is the variable nitrogen and protein content due to sex and age within the developmental stage and the feeding and breeding methods [38,39].

### 3.2. Colour Measurement

All the pancakes with the addition of insect meal were darker than the control sample (an increase in the L* parameter value), and the greater the addition of each insect meal type was, the darker the colour was. The most intense colour changes were noted for pancakes with *T. molitor* added. The addition of all powdered insects resulted in an increased intensity of the red colour (an increase in the parameter a* value) of the pancakes compared with the control sample. The greatest intensity of this colour was observed in pancakes with a 30% addition of *T. molitor*. The addition of the *A. diaperinus* had the smallest effect on the increase in the red colour intensity. None of the insect meal additives had a significant effect on a change in the yellow colour of the pancakes (the b* parameter value). The obtained browning index values demonstrated no statistically significant differences between the pancakes. The results of the colour measurement are provided in Table 4.

The difference in the test pancake colours was compared with those of the control pancakes. Based on the ΔE results obtained from mean values of L*, a*, and b*, it can be assumed that a standard observer notices a clear difference between the colour of the pancakes with the addition of *A. diaperinus* and *A. domesticus* in amounts of 10% and 20% for each of these additives. As for the pancakes with a 30% addition of *A. diaperinus* and *A. domesticus*, there was a clear impression of two different colours. Similar results were obtained for the pancakes with 10%, 20%, and 30% additions of *T. molitor*. The smallest difference between the colours was noted for the pancakes with a 10% addition of *A. domesticus*, while the greatest colour difference was noted for the pancakes with 30% *T. molitor* added. For all the test pancakes, the difference in colour was clearly noticeable to an average observer.

In the current study, all the pancake variants were darker than the control sample, which is consistent with the results of a study conducted by Gaglio et al. [40]. In studies by other authors, cereal products with the addition of both *A. domesticus* and *T. molitor* at 5% and 10% levels exhibited the values ΔE > 3 as compared with the control sample, which resulted in the differences in colours being noticeable to consumers [41,42]. In the current study, all the variants were noticeable to the consumer (ΔE > 3). The differences in colours noted by the testers determined the appearance assessment results and shaped the overall preferences of the test group. The excessively dark colour of the pancakes received no high acceptability, which consequently translated into a lowered appearance rating. A study by Garciá-Segovia et al. demonstrated that the addition of *A. domesticus* and *T. molitor* to bread resulted in lower values of the a* parameter as compared with the control sample [41]. Bread baked with a 10% addition of insect powder showed no statistically significant differences in terms of the parameter b* value as compared with the control sample. In the current study, for all the pancake variants, non-significant changes in the parameter b* value were noted. As regards the parameter a*, increased intensity of the red colour was noted for all variants, with the greatest influence demonstrated for the variant with a 30% addition of the *T. molitor*. A previous study (Zielińska et al., 2021) noted a greater increase in the parameter a* value in muffins with the addition of crickets of the *Gryllodes sigillatus* species as compared with the samples containing *T. molitor*. The same trend was observed for the pancakes with the addition of the *T. molitor* and *A. domesticus* species that were tested. This may be an effect of the higher protein content in the *A. domesticus* meal; furthermore, protein is a substrate in the Maillard reaction, which is responsible for increasing the intensity of the red colour. The key factor determining the changes in the colour due to the Maillard reaction is the presence of monosaccharides. In breads, they are used up in the fermentation process, while in pancakes and muffins, they remain present, thus affecting both the intensity of the red colour and browning [18].

Tukey’s test for homogeneous groups demonstrated that a 30% addition of *T. molitor* and the addition of *A. domesticus* in each case had a significant effect on the pancake hardness as compared with the control sample. The greatest differences in cohesiveness in relation to the control sample were exhibited by pancakes with *A. domesticus* added. The cohesiveness value increased with an increase in the insect powder content. The pancakes with a 10% and 30% addition of *T. molitor* meal were statistically significantly more cohesive than the control sample. The results of measurements of the hardness and cohesiveness of the pancakes with insect meal added are provided in Table 5.

In terms of hardness, pancakes with the addition of *T. molitor* in amounts of 20% and 10% did not statistically significantly differ from the control sample. However, pancakes with 10% *T. molitor* added demonstrated no such difference. None of the pancake variants with the addition of *A. diaperinus* differed statistically significantly from the control sample in terms of hardness. The pancakes with the *A. domesticus* added were characterised by the greatest hardness. All pancake variants with *A. domesticus* meal added were statistically significantly harder than the control sample. With the addition of *A. domesticus* meal, the pancake hardness level increased.

Khatun et al. (2021) concluded that the hardness of chapati flatbreads increased with an increase in the *A. domesticus* meal content [43]. The gelatinisation temperature did not change significantly up to a 10% substitution of wheat flour and decreased with a 15% addition of *A. domesticus* meal. A downward trend in the gelatinisation temperature was also described by Indriani et al. in a study involving a 20% addition of an insect (*Patanga succincta*) to rice flour [44]. The replacement of wheat flour with insect meal reduced the starch content in the composition. Water was absorbed by proteins and fibre instead of being used for starch gelatinisation [44,45]. An increased amount of proteins, lipids, and fibres dilutes the starch content in the flour and reduces starch swelling and gelation during heat treatment, thus reducing the cohesiveness and affecting the texture [46,47]. A slight substitution of wheat flour with insect meal results in a favourable reduction in hardness, while an excessive amount results in an unfavourable increase in hardness that reduces the acceptability of the product. The amount of substitution at which the hardness increases is primarily determined by the amount of water in the product. Osimani et al. concluded that the properties of wheat flour did not change up to 10% enrichment with meal from *A. domesticus*; moreover, a further increase in the addition of insect meal had a significant, adverse effect on the structure [48]. However, a study conducted by González et al. did not demonstrate that the addition of meal from insects (*A. domesticus* and *T. molitor)* resulted in an increased hardness of the breadcrumb [49]. Completely different study results were presented by Roncolini et al., who demonstrated that the addition of *T. molitor* to bread in amounts of 5% and 10% resulted in reduced hardness [50]. It was also reported by Severini et al. that the addition of *T. molitor* to cereal snacks significantly reduced the hardness of test products [51]. The best effects in terms of bread texture and hardness were obtained by Kowalski et al. from *T. molitor* meal [52]. In the current study, the addition of 30% *T. molitor* meal and 10%, 20%, and 30% of *A. domesticus* meal significantly increased the hardness. However, no such tendency was noted for *A. diaperinus*. It is likely that the direction of changes in the hardness and cohesiveness of products with the addition of edible insects is not determined by the proportion of insect meal but primarily by the insect species. This was confirmed by a study by Zielińska et al., which found that the addition of *A. domesticus* had a greater effect on the texture than the addition of *T. molitor* [18]. In the current study, for each insect species, a sample with 30% substitution was characterised by the greatest hardness.

### 3.3. Acceptability

The results obtained from variance analysis (ANOVA) showed that the amount of the additive had a statistically significant effect on all the distinguishing features of the sensory evaluation, while the additive type had no such effect. The type and amount of the additive had a statistically significant effect only on the pancake structure. The values marked with the same symbols in a particular group were characterised by similar values within the sensory characteristic being tested. Regarding the characteristics under assessment, a significant parameter that affected the rating value was the amount of the additive and not its type. The respondents gave the highest rating to the pancakes with no insect meal added. An increase in the added meal content reduced the ratings of all the parameters being assessed by the assessment panel. The results are provided in Table 6.

Regarding appearance, the highest scores were noted when assessing the control sample. Of all the pancakes with insect meal added, the samples with a 10% insect content were rated the highest. An exception was the pancakes with *A. diaperinus* added, where significantly different appearance acceptability results were obtained for the 10% and 20% additives. The respondents gave the highest rating to the aroma of pancakes with a 10% addition of *T. molitor*. As for the 20% addition of *A. domesticus*, a wide divergence of results was noted, which suggests that the perceptible flavour of *A. domesticus* is a very positive aspect for some respondents while being very negative for others. However, considering the low ratings obtained by the pancakes with a 30% addition, it can be assumed that the preference is largely determined by the intensity of the aroma. As regards *T. molitor* and *A. diaperinus*, the acceptability of their aroma decreases with an increase in the insect powder content in the pancakes. As for the flavour, the testers rated the control sample the highest. In contrast, pancakes with an addition of insects obtained increasingly lower scores as the addition of powdered insects increased.

The texture was rated the lowest for the sample of pancakes with a 30% addition of *A. domesticus* (Cr30). Similar results were obtained for pancakes with a 10% and 20% addition of *A. diaperinus*. The control sample was rated the highest. The pancakes containing 30% insect meals were rated the lowest for each insect species. In the study on overall acceptability, the highest score was obtained for the control sample. However, regarding the pancakes with insects added, the overall acceptability decreased with an increase in the insect meal content. The species of the added insect had no significant effect on the result in terms of acceptability. Of all the pancake variants, the lowest score for the overall acceptability was obtained by the sample with a 30% addition of *A. domesticus*. The result of Kowalski et al. proved that sponge cakes without insects obtained the highest acceptability. The variants with *T. molitor* obtained lower acceptability independently of the amount of meal [53].

As for the analysis of the distinguishing qualitative features affecting the acceptability of new insect meal-based products, very important elements were the structure and texture of the test samples. It appeared that the addition of insect meal had a significant effect on the texture and, indirectly, on the overall acceptability as well. In particular, the acceptability of texture for the *A. domesticus* differed statistically significantly from the rating of the pancakes with the addition of the *T. molitor* and the *A. diaperinus*. This may be due to the fact that only the *A. domesticus* was in its non-larval form. Of all the test variants, products with the *A. domesticus* added contained the most chitin derived from the insect’s exoskeleton. Pancakes with a 30% addition of *A. domesticus* meal were hard and slightly rubbery, which consequently determined the lowered rating of the texture. The greater the proportion of *A. domesticus* meal, the lower the rating of the texture was. However, only in the case of the variant with a 30% *A. domesticus* content were statistically significant differences noted in the ratings in relation to the control sample. Barton et al. obtained completely different results. Consumption of the protein preparation with a 30% addition of powdered cricket did not reduce the evaluation of sensory features compared with the control sample [54].

Based on the organoleptic evaluation results, it can be concluded that all the tested characteristics were rated the highest for the control pancakes. Each of the additives used reduced the overall acceptability, and with an increase in the amount of each additive, the ratings for the tested pancakes decreased. As demonstrated in a study by Grossmann et al., the sensory profiles of insects change significantly when exposed to processing [55]. Depending on the type and conditions of heat treatment and the product used, the nature of the flavour and aroma profile of the insects themselves can vary considerably. The degree of acceptability of insects in food products is determined by multiple factors and not exclusively by the insect species. A study by Adamek et al. demonstrated that the method for processing both the insects themselves and the product in which they are used significantly changed the aroma profile, i.e., one of the elements determining the degree of acceptability of a particular product with an addition of edible insects [56]. For this reason, the degree of acceptability of the same insect species is probably different depending on the main carrier.

In order to determine which of the factors under consideration had the strongest effect on the overall acceptability, equations were determined based on the physicochemical data analysed in the study for all variants. The obtained multiple equations of overall acceptability are provided in Table 7.

Multiple regression analysis showed that for the pancakes with insect meal added, the aroma proved to be insignificant and had no effect on the overall acceptability of the test pancakes. The R^2^ correlation coefficient value for the test samples was high and amounted to: R^2^_Mw_ = 0.87, R^2^_Bw_ = 0.87, and R^2^_Cr_ = 0.89, which means that the generated model explains almost 90% of the variability of the dependent variable. The obtained results indicate that the level of acceptability was a result of the predictors taken into account in the regression equation, of which the flavour was the most important for the overall acceptability. The other variables took positive values in the determined equation, which suggests that their presence contributed to the overall level of acceptability of the pancakes with insects added. Only 10% of the variability was determined by other, non-analysed parameters. In contrast, Sogari et al. obtained results that showed that texture and appearance were the main components that reduced acceptability rather than taste. However, that study was conducted with whole insects, and the carrier was jelly. It is, therefore, possible that the grainy texture was an extremely undesirable factor in jellies but not in general food products [57].

For food products, a key element affecting a product’s quality is its sensory characteristics. In order for a product to have a chance of becoming a regular component of a population’s diet, it needs to be acceptable in terms of certain descriptors, for example, the appearance, flavour, aroma, and texture. A regression analysis demonstrated that flavour was the deciding determinant influencing the overall acceptability. The greater the addition of insect meal, the lower the rating of the flavour was. For example, a consumer assessment conducted in Hungary showed that the flavour of oat biscuits with a 5% addition of *A. domesticus* meal was rated similarly to the control sample (with no *A. domesticus* added). On the other hand, biscuits with a higher *A. domesticus* content (10 and 15%) obtained statistically significantly lower scores in terms of flavour [58]. A similar trend was also observed in the current study. On the other hand, Çabuk and Yılmaz tested samples of pasta with the addition of edible insects. In this case, the flavour was rated inferior to the control sample. Pasta enriched with grasshopper imago was rated slightly lower than pasta containing *T. molitor* larvae, which indicates a dependence of acceptability not only on the mere change in the recipe and the insect addition but also on the insect species and form [59]. In a study, Mandolesi et al. (2022) showed that for consumers who did not show a negative attitude, the acceptability of individual insect species in food varied. However, despite the different attitudes and conditions of perceiving edible insect-based food, the medium is still one of the key factors that determine the degree of liking [60].

The current analysis also demonstrated that the flavour was rated the lowest for *A. domesticus* (imago). However, what was crucial to the flavour was the amount of insect added. A study by Osimani et al. (2018) conducted in Italy confirmed that with an increase in the content of *A. domesticus*, the scores for the bread flavour decreased [48].

The results obtained by Tan et al. indicated that the acceptance of insects as food is a more important factor influencing acceptance than sensor-liking. Studies have shown that consumers expect a dish with insects to taste worse, even if it looks identical to the conventional version [61]. It can therefore be assumed that consumers, having obtained information about the presence of insects in a product or a dish, have a tendency to judge the taste as inferior.

### 3.4. Attitudes towards the Consumption of Pancakes with Added Insect Meal

Even though entomophagy is common in many regions of the world and is part of traditional cuisine, for Western societies, insects are a new ingredient [62,63,64]. According to Shiv and Fedorikhin, when unfamiliar foods are concerned, relatively frequent ambivalent attitudes, including both positive and negative components, are to be expected [65]. Ambivalence can manifest itself when there is a conflict between curiosity and fear of the consequences of consumption, and between the appearance and the knowledge of the nutritional value of the product, i.e., a conflict between desire and avoidance. In the current study, ambivalent attitudes accounted for a small proportion. As many as 80% of respondents were not afraid to try pancakes prepared with the addition of insects. This suggests that they did not expect negative health consequences from their consumption. Additionally, these results might have been influenced by the fact that the testers trying the products concerned were informed that the insects were farmed and not collected from the wild. The exact results of a survey on consumer opinions regarding the consumption of insects following the tasting of the pancakes are provided in Table 8.

The respondents strongly differentiated between the pancakes in terms of their visual attractiveness. The taste of the pancakes, however, was not a positive surprise for the respondents. The question about the taste received the greatest number of responses, indicating an ambivalent attitude compared with the remaining questions. According to Martins and Pliner, the readiness to taste insects for the first time is conditioned by interest and a low level of disgust. The expectation of a positive taste experience becomes a driving factor for entomophagy later [66]. The main factors at the later stages of entomophage implementation include price, availability, taste, and the ability to use insects in culinary practice [67,68,69]. The results obtained by Tan and House show that people who do not eat insects, who come from countries with a highly developed tradition of entomophagy, have knowledge about insect consumption and perceive insects as part of the nutrition of their community [69]. Additional factors in the success of introducing insects into the diet are eating practices, including eating ready-to-eat vegetarian meals, the level of attachment to eating traditional meals, and factors related to obtaining food [3].

In the current study, the majority of respondents were willing to recommend that others try this product. However, over half of them showed a negative attitude toward the prospect of including insects in their diets. In a study conducted by Ros-Baró and Sánchez-Socarrás et al. (2022), more than 80% of participants indicated that they did not eat insects before and did not want to include insects in their regular diet. Disgust, followed by a lack of habit and concern for safety, were the main reasons participants cited as justification for not being interested in eating insects [70]. Halloran and Flore noted that even among future chefs, there is a very low level of knowledge related to the use of insects in food recipes to emphasize the taste of insects [71]. This was the second most frequently indicated barrier against the use of insects in modern gastronomy. Among the respondents, 47% described disgust as the main barrier. It was a special study group, where as many as 76% of participants had tried insects at least once before. Additionally, some of the respondents came from countries with a widespread tradition of eating insects. Significant obstacles in the dissemination of entomophagy in gastronomy were also the low availability of products, concerns about food safety, the association of entomophagy with poverty, and the current high prices of insects [71].

Nevertheless, this does not prevent almost half of the respondents from being ready to try other products with insects added. In a study conducted by Tuccillo et al. among Italian consumers, a positive attitude towards entomophagy was demonstrated by 41% of respondents, while a negative attitude was demonstrated by 27% of respondents [72]. An ambivalent attitude was shown by 32% of respondents. Based on the sensory assessment, the authors concluded that a low level of insect visibility was preferred. After tasting, the respondents demonstrated a more positive attitude towards the consumption of insects in the imago form than in the larval form. The results of the current study show a different trend. On the other hand, in a Belgian analysis, more than 65% of respondents showed an entomophagy-rejecting attitude [62]. It is worth noting, however, that studies conducted so far have confirmed the strong adverse effect of the fear of trying new foods and the willingness to consume insects and/or insect-based dishes on the overall perception of entomophagy [11]. Barton et al. showed that after tasting, the subjects showed more positive attitudes towards entomophagy—the level of perception towards entomophagy as an unnatural phenomenon decreased. In addition, disgust was reduced and the number of declarations to include insects in the diet increased. The authors note, however, that these results do not necessarily translate into future eating behaviour. However, in the case of concerns about the presence of pathogenic microorganisms and toxins, the attitudes of the participants did not change significantly [54]. The same result was shown in a study by Lensvelt and Steenbekkers. However, the effect was greater for people who had tried insects before [73]. Menozzi et al. indicated that the reason for the reluctance to include insects in the diet is the fear of the negative opinion of the elderly in the family, and friends and society who may perceive insects as inedible [74]. These concerns are confirmed by Myers and Pettigrew, who found a very low level of awareness among the elderly about the nutritional and environmental benefits of entomophagy. Additionally, insects were often associated with dirt, poverty, and disgust [75]. Therefore, the results concerning attitudes towards entomophagy differed significantly depending on whether the study was a questionnaire survey or a questionnaire survey combined with tasting. In addition to increasing the acceptability of entomophagy, a willingness to consume edible insects and the verification of their acceptable form are also necessary. Disgust is a psychological factor that acts as a barrier to entomophagy. According to Ruby et al., disgust is mainly based on the knowledge of potential foods and not on their sensory properties, i.e., characteristics assessed through the senses (taste, sight, smell, touch, and hearing) [10]. It is also important to note that since the feeling of disgust in particular individuals is determined by multiple factors, it varies with respect to their choices and behaviours and is strongly influenced by cultural and social factors [11,76]. The results obtained by Modlinska et al. (2020) proved that information about insects determines preference more than appearance or odour. Foods labelled as insect-containing were tasted later, faster, and in smaller amounts than food labelled insect-free, even if it did not contain elements suggesting the presence of insects. General neophobia levels correlated with the latency to pick up food. The participants had not been informed that the experiment was about tasting insect-based products, so their attitudes and expectations did not influence their responses [77]. Research conducted by Gmuer et al. (2016) indicated that consumers expect a large number of different negative emotional experiences before the consumption of snacks containing cricket. This occurrence may affect the objective evaluation of organoleptic parameters [78]. Food preferences are formed in childhood. According to Skinner et al. and De Cosmi et al., encouraging entomophagy should be implemented in children because socio-cultural factors constitute a serious barrier against insect consumption among adults [79,80].

On the other hand, an analysis conducted for two extremely different populations (German and Chinese) demonstrated significant discrepancies in the degree of entomophagy acceptability. In Germany, a positive attitude towards the willingness to eat insects was considerably greater for processed than for unprocessed food products, while in China, no such difference was noted. Significant differences were also noted in the perception of the nutritional value, flavour and knowledge, and social acceptability of entomophagy, with more positive attitudes reported by the Chinese compared with the Germans [11]. However, as indicated by the current study, even in the case of powdered insects, Polish testers would not be willing to introduce insects into their diets.

Consumers differ significantly in their willingness to try a new product. For each type of product found on the market, there are so-called pioneering consumers and early followers. Others accept innovative products with a significant delay. After a slow start, more and more people then begin to accept a new product. It can therefore be assumed that in Western countries, only pioneers and early followers are ready to introduce insects into their diets [81]. Mandolesi et al. (2022) classified consumers who consumed insects into three groups. The most numerous were traditional consumers who believed that insects should not be an ingredient of well-known, traditional dishes. They showed a higher level of aversion to novelty and entomophagy. In another study among the potential carriers for enrichment with edible insects, participants most often indicated flour products (Ros-Baró et al., 2022).

The results of the current study and other studies suggest that Western society is currently at the stage of learning about insects as a source of food and is interested in them. These products are not yet known well enough to become a regular part of the diet.

The current study was focused exclusively on young people, which does not fully reflect the acceptability of entomophagy in society. Moreover, only one recipe variant was tested. It is possible that a modification of the basic pancake recipe would alter the results. All of the pancake samples with the addition of edible insects were rated against the control sample, which provides an opportunity to objectively compare the effect of insect addition on individual characteristics. This helped to exclude the negative ratings of organoleptic characteristics for the testers who disliked the pancakes. Considering that many young respondents showed positive attitudes towards entomophagy after trying the pancakes, it can be concluded that there is a great opportunity for the development of the edible insect market throughout Europe. Further research is necessary, taking into account different ingredient ratios and a more varied insect meal content. It is possible that the spread of entomophagy will increase over time as consumers become accustomed to the previously unfamiliar flavour of edible insects. The advantage of the study is that consumer attitudes were measured after tasting products with the addition of insects, which makes their declarations more objective and closer to actual behaviour than theoretical considerations concerning attitudes toward entomophagy. The research was conducted on a small group of young people. More research is needed, taking into account different age groups. Future research should focus on the specific motives of attitudes and take into account factors such as the frequency of previous insect consumption, as this significantly determines the level of taste preference.

## 4. Conclusions

Most of the attitudes towards entomophagy and the consumption of pancakes with the addition of edible insects were positive, even though the majority of the respondents were deterred from trying the test samples due to the addition of insect meal. The key factor influencing the overall sensory acceptability was the flavour. The other factors, including the texture, aroma, and appearance, had a considerably lesser effect on the overall acceptability level. Pancakes with insects were well accepted by consumers, provided that a small (10%) addition of insect meal was used, thus resulting in an acceptability level similar to that for conventional wheat pancakes. However, the overall acceptability level in relation to the control sample decreased with an increase in the insect content. With an increase in the insect meal content, the pancake lightness (L*) and hardness decreased. The findings of the current study indicated that the acceptability of insect-containing products is primarily determined by the amount of insect meal added and, to a much lesser extent, by the insect species. The attitude survey was combined with tasting, which ensured a greater reliability of the data than in questionnaire surveys. The use of wheat pancakes as a base has been proposed as part of a wider project involving the use of edible insects in other food products. The results of the current study may provide guidance for the food industry regarding the production of new high-protein foods and functional foods based on edible insects. The obtained acceptability results indicated that insect-based products can now be introduced in Western society.

## Figures and Tables

**Table 1:** Pancake ingredients.

Ingredient	Control g	10% Insect Meal g	20% Insect Meal g	30% Insect Meal g
Milk	100	100	100	100.0
Eggs	30	30	30	30
Oil [g]	11	11	11	11
Salt	1	1	1	1
Wheat flour	92	83	65	50
Insect meal	0	9.2	18.4	27.6

**Table 2:** Statements on the consumption of edible insects.

Statement
1. I was afraid of trying the pancakes.
2. All pancake variants looked equally appetizing.
3. The addition of insect meal discouraged me from trying the pancakes.
4. The flavour of pancakes with insects added positively surprised me.
5. I would like to try other products with an addition of insects.
6. I might consider incorporating pancakes with the addition of insects into my diet.
7. I would recommend others try pancakes with an addition of insect meal.

**Table 3:** Nutritional value of 100 g pancakes.

Sample	Designation	Energy kcal	Protein g	Fat g	Carbohydrates g	Ash g	Fibre g	Moisture g
Control	C	255.32 ^c^ ± 3.1	8.13 ^b^ ± 0.19	7.93 ^a^ ± 0.1	34.23 ^a^ ± 0.88	0.98 ^a^ ± 0.05	1.13 ^a^ ± 0.06	40.32 ^a^ ± 1.22
*T. molitor*	10% Mw	263.74 ^b^ ± 2.4	9.29 ^a^ ± 0.54	9.15 ^b^ ± 0.12	29.39 ^b^ ± 0.76	0.96 ^a^ ± 0.01	1.26 ^b^ ± 0.01	40.68 ^a^ ± 0.94
(larve)	20% Mw	269.66 ^b^ ± 2.3	10.53 ^bc^ ± 0.68	10.84 ^b^ ± 0.52	24.33 ^c^ ± 0.45	0.96 ^a^ ± 0.01	1.35 ^b^ ± 0.01	43.02 ^a^ ± 0.95
	30% Mw	275.86 ^d^ ±2.6	11.73 ^c^ ± 0.38	12.37 ^e^ ± 0.22	19.65 ^cd^ ± 0.66	0.97 ^b^ ± 0.05	1.46 ^c^ ± 0.04	41.47 ^b^ ± 1.05
*A. diaperinus*	10% Bw	260.87 ^a^ ± 1.9	9.98 ^a^ ± 0.16	8.70 ^b^ ± 0.37	29.73 ^bc^ ± 0.43	0.91 ^b^ ± 0.02	1.22 ^c^ ± 0.05	40.73 ^ab^ ± 1.29
(larve)	20% Bw	266.67 ^b^ ± 1.7	11.99 ^c^ ± 0.85	9.96 ^bc^ ± 0.45	25.05 ^c^ ± 0.33	0.87 ^c^ ± 0.04	1.27 ^c^ ± 0.05	40.63 ^ab^ ± 1.20
	30% Bw	260.87 ^a^ ± 2.1	14.00 ^d^ ± 0.29	11.10 ^e^ ± 0.1	20.55 ^ac^ ± 0.77	0.86 ^c^ ± 0.04	1.33 ^c^ ± 0.04	40.84 ^bc^ ± 0.61
*A. domesticus*	10% Cr	260.87 ^a^ ± 0.9	10.48 ^bc^ ± 0.44	8.50 ^b^ ± 0.19	29.64 ^e^ ± 0.55	0.99 ^a^ ± 0.05	1.32 ^a^ ± 0.01	39.09 ^d^ ± 0.46
(imago)	20% Cr	260.87 ^a^ ± 2.7	13.05 ^e^ ± 0.34	9.54 ^bc^ ± 0.29	24.69 ^ce^ ± 0.88	1.12 ^b^ ± 0.04	1.51 ^d^ ± 0.01	40.53 ^ac^ ± 0.65
	30% Cr	260.87 ^a^ ± 3.2	15.66 ^ef^ ± 0.66	10.46 ^d^ ± 0.18	19.94 ^cd^ ± 0.26	1.21 ^b^ ± 0.06	1.70 ^e^ ± 0.08	43.03 ^a^ ± 0.92

^a–f^ Mean values followed by the same letter are not significantly different according to *p* < 0.05.

**Table 4:** Lightness, a*, and b* value intensities of pancakes prepared with different ratios of *A. diaperinus*, *T. molitor*, and *A. domesticus*.

Sample	Designation	L*	a*	b*	ΔE	Bl
Control	C	48.89 ^a^ ± 2.21	−0.49 ^a^ ± 0.25	24.15 ^abc^ ± 1.06		64.13 ^a^ ± 6.78
*T. molitor*	10% Mw	55.90 ^cd^ ± 1.20	2.14 ^bc^ ± 0.31	23.47 ^abc^ ± 1.83	3.83	55.21 ^a^ ± 5.38
(larve)	20% Mw	57.68 ^d^ ± 1.54	5.66 ^e^ ± 0.82	24.54 ^bc^ ± 2.33	4.39	60.86 ^a^ ± 7.00
	30% Mw	67.23 ^e^ ± 2.19	8.35 ^f^ ± 0.79	25.31 ^c^ ± 1.22	9.41	54.93 ^a^ ± 3.56
*A. diaperinus*	10% Bw	52.51 ^bc^ ± 1.87	1.74 ^b^ ± 0.18	24.04 ^abc^ ± 0.82	7.39	61.17 ^a^ ± 5.44
(larvae)	20% Bw	52.48 ^abc^ ± 2.43	2.98 ^cd^ ± 0.23	23.74 ^abc^ ± 1.29	10.2	62.09 ^a^ ± 5.69
	30% Bw	57.68 ^d^ ± 1.54	3.82 ^d^ ± 0.60	23.72 ^abc^ ± 1.11	19.98	55.97 ^a^ ± 3.99
*A. domesticus*	10% Cr	50.70 ^ab^ ± 2.63	1.36 ^b^ ± 0.18	21.66 ^a^ ± 1.52	3.2	55.64 ^a^ ± 6.03
(imago)	20% Cr	51.16 ^ab^ ± 0.92	3.52 ^d^ ± 0.75	22.34 ^ab^ ± 1.45	4.19	60.24 ^a^ ± 4.90
	30% Cr	56.94 ^d^ ± 1.61	5.06 ^e^ ± 0.68	25.56 ^c^ ± 1.66	9.36	64.08 ^a^ ± 7.65

^a–f^ Mean values followed by the same letter are not significantly different according to *p* < 0.05.

**Table 5:** Additive’s influence on deformation at hardness—the cohesiveness of pancakes (TPA test).

Sample	Designation	Hardness [N]	Cohesiveness [N]
Control	C	1.670 ^a^ ± 0.044	0.760 ^a^ ± 0.040
*A. diaperinus*	10% Bw	1.664 ^a^ ± 0.015	0.753 ^a^ ± 0.031
(larvae)	20% Bw	1.667 ^a^ ± 0.039	0.760 ^a^ ± 0.020
	30% Bw	1.677 ^a^ ± 0.035	0.760 ^a^ ± 0.040
*T. molitor*	10% Mw	1.680 ^a^ ± 0.034	0.780 ^ab^ ± 0.020
(larvae)	20% Mw	1.677 ^a^ ± 0.026	0.760 ^a^ ± 0.040
	30% Mw	1.906 ^b^ ± 0.098	0.793 ^ab^ ± 0.031
*A. domesticus*	10% Cr	2.027 ^bc^ ± 0.108	0.827 ^ab^ ± 0.031
(imago)	20% Cr	2.112 ^c^ ± 0.065	0.833 ^ab^ ± 0.023
	30% Cr	2.844 ^d^ ± 0.026	0.860 ^b^ ± 0.040

^a–d^ Mean values followed by the same letter are not significantly different according to *p* < 0.05.

**Table 6:** The results of pancake consumer’s organoleptic evaluation.

Sample	Designation	Taste	Odour	Appearance	Structure	Preference
Control	C	7.09 ^a^ ± 2.86	7.36 ^ab^ ± 2.90	8.27 ^ce^ ± 2.00	7.22 ^cef^ ± 2.66	7.29 ^ab^ ± 2.78
*T. molitor*	10% Mw	6.06 ^ae^ ± 2.61	7.70 ^b^ ± 2.66	6.67 ^abce^ ± 2.60	6.15 ^abc^ ± 2.82	6.23 ^abde^ ± 2.61
(larve)	20% Mw	4.70 ^bde^ ± 3.07	6.66 ^abc^ ± 3.05	6.45 ^abc^ ± 3.03	6.17 ^abc^ ± 2.44	5.08 ^cde^ ± 2.65
	30% Mw	3.25 ^bc^ ± 2.98	5.96 ^abc^± 3.14	5.17 ^ad^ ± 2.95	5.27 ^abd^ ± 2.93	3.35 ^fgh^ ± 3.08
*A. diaperinus*	10% Bw	5.83 ^ade^ ± 2.75	6.30 ^abc^ ± 2.61	6.77 ^abce^ ± 2.59	6.67 ^bcef^ ± 2.83	5.78 ^acde^ ± 2.94
(larvae)	20% Bw	4.29 ^bcd^ ± 2.58	6.04 ^abc^ ± 2.85	5.83 ^abd^ ± 2.58	5.47 ^abd^ ± 2.90	4.22 ^cgh^ ± 2.81
	30% Bw	3.11 ^bc^ ± 3.01	5.07 ^c^ ± 3.20	4.54 ^ad^ ± 2.88	4.99 ^ad^ ± 2.84	3.09 ^fg^ ± 2.75
*A. domesticus*	10% Cr	6.60 ^a^ ± 2.49	6.75 ^abc^ ± 2.42	6.20 ^abc^ ± 2.73	6.42 ^abce^ ± 2.53	6.73 ^abe^ ± 2.35
(imago)	20% Cr	4.81 ^bde^ ± 2.56	7.18 ^abc^ ± 8.20	4.67 ^ad^ ± 2.61	5.58 ^abc^ ± 2.46	4.82 ^cdh^ ± 2.31
	30% Cr	2.71 ^c^ ± 2.56	5.34 ^ac^ ± 2.93	3.70 ^d^ ± 3.31	3.83 ^d^ ± 2.95	2.51 ^f^ ± 2.49

^a–h^ Mean values followed by the same letter are not significantly different according to *p* < 0.05.

**Table 7:** Multiple equations of overall pancake acceptability.

Type of Additive	Regression Equation	R^2^	F
Control	y = 0.032x_1_ + 0.691x_2_ − 0.064x_3_ + 0.330x_4_	0.92	47.402
*A. diaperinus* (in general)	y = 0.160x_1_ + 0.687x_2_ + 0.099x_4_	0.87	65.972
*T. molitor* (in general)	y = 0.141x_1_ + 0.691x_2_ + 0.081x_4_	0.87	82.495
*A. domesticus* (in general)	y = 0.237x_1_ + 0.545x_2_ + 0.162x_4_	0.89	79.672

R^2^—determination coefficient, F—statistic value, y—preference; x_1_—structure; x_2_—taste; x_3_—odour; x_4_—appearance.

**Table 8:** Consumers showing negative, ambivalent, or positive attitudes.

Components of Attitudes	Positive	Ambivalent	Negative
1. I was afraid of tasting cakes	13.33%	6.67%	80.00%
2. All variants of the pancakes looked equally delicious	33.33%	5.00%	61.67%
3. The addition of insect meal discouraged me from tasting the pancakes	63.33%	6.67%	30.00%
4. The taste of the pancakes with the addition of edible insects surprised me positively	43.33%	21.67%	35.00%
5. I would like to try other products with the addition of insects	48.33%	20.00%	31.67%
6. I could include insect pancakes in my diet	58.33%	15.00%	26.67%
7. I would recommend that others try pancakes with the addition of insect meal	65.00%	15.00%	20.00%

## Data Availability

The data presented in this study are available on request from the corresponding author.
